# Severe Nephrotoxic Nephritis following Conditional and Kidney-Specific Knockdown of Stanniocalcin-1

**DOI:** 10.1371/journal.pone.0138440

**Published:** 2015-09-22

**Authors:** Luping Huang, Yahuan Lou, Huiming Ju, Lin Zhang, Jenny Szu-Chin Pan, April Ross, Yuxiang Sun, Luan D. Truong, David Sheikh-Hamad

**Affiliations:** 1 Division of Nephrology/Department of Medicine, Baylor College of Medicine, Houston, TX, United States of America; 2 Dental Branch, University of Texas Health Science Center, Houston, TX, United States of America; 3 College of Veterinary Medicine, Yangzhou University, Yangzhou 25009, Jiangsu, P. R.China; 4 Center of General Surgery, Chengdu General Hospital of Chengdu Military Area Command, Chengdu, P. R. China; 5 Children’s Nutrition Research Center, Baylor College of Medicine, Houston, TX, United States of America; 6 Pathology and Laboratory Medicine, Methodist Hospital/Weill Cornell Medical College, Houston, Texas, United States of America; UCL Institute of Child Health, UNITED KINGDOM

## Abstract

**Background:**

Inflammation is the hallmark of nephrotoxic nephritis. Stanniocalcin-1 (STC1), a pro-survival factor, inhibits macrophages, stabilizes endothelial barrier function, and diminishes trans-endothelial migration of leukocytes; consistently, transgenic (Tg) overexpression of STC1 protects from nephrotoxic nephritis. Herein, we sought to determine the phenotype of nephrotoxic nephritis after conditional and kidney-specific knockdown of STC1.

**Methods:**

We used Tg mice that, express either STC1 shRNA (70% knockdown of STC1 within 4d) or scrambled shRNA (control) upon delivery of Cre-expressing plasmid to the kidney using ultrasound microbubble technique. Sheep anti-mouse GBM antibody was administered 4d after shRNA activation; and mice were euthanized 10 days later for analysis.

**Results:**

Serum creatinine, proteinuria, albuminuria and urine output were similar 10 days after anti-GBM delivery in both groups; however, anti-GBM antibody delivery to mice with kidney-specific knockdown of STC1 produced severe nephrotoxic nephritis, characterized by severe tubular necrosis, glomerular hyalinosis/necrosis and massive cast formation, while control mice manifested mild tubular injury and crescentic glomerulonephritis. Surprisingly, the expression of cytokines/chemokines and infiltration with T-cells and macrophages were also diminished in STC1 knockdown kidneys. Staining for sheep anti-mouse GBM antibody, deposition of mouse C_3_ and IgG in the kidney, and antibody response to sheep IgG were equal.

**Conclusions:**

nephrotoxic nephritis after kidney-specific knockdown of STC1 is characterized by severe tubular and glomerular necrosis, possibly due to loss of STC1-mediated pro-survival factors, and we attribute the paucity of inflammation to diminished release of cytokines/chemokines/growth factors from the necrotic epithelium.

## Introduction

The mammalian pro-survival protein STC1 is expressed in many tissues and organs including the kidneys [1]. It is released to the extracellular milieu [2], and binds to a cell-surface protein [3], followed by internalization and targeting to the inner mitochondrial membrane [4,5]. It is defined as a paracrine/intracrine protein; i.e., intracellular-acting, extracellular signaling protein [6]. In cultured endothelial cells, STC1 diminishes superoxide generation, inhibits cytokine-induced signaling through Jun-N-terminal kinase (JNK) and Nuclear Factor-kappaB (NF-κB), and preserves endothelial barrier function [7]. STC1 inhibits macrophages through a number of mechanisms that include: suppression of superoxide generation through increasing uncoupling proteins [8]; inhibition of macrophage response to chemoattractants [9] and migration across endothelial cells [10].

These observations suggested that STC1 may have potent anti-inflammatory and cytoprotective effects. Indeed, STC1 transgenic mice, which display elevated serum levels of rSTC1 and preferential expression of the transgene in macrophages and endothelial cells, are protected from anti-GBM glomerulonephritis [11]. The contribution of kidney-derived STC1 to the anti-inflammatory and cytoprotective effects it confers is unknown, and we hypothesized that kidney-specific knockdown of STC1 will aggravate inflammation in the setting of nephrotoxic nephritis.

Kidney-specific knockdown of STC1 was achieved as we recently described [12], using STC1 shRNA Tg and scrambled shRNA Tg mice. Delivery of Tie2-Cre to the kidney using ultrasound microbubbles, removes a floxed reporter (PGK-EGFP), permitting the expression of shRNA and kidney-specific knockdown of STC1 in STC1 shRNA Tg kidneys (70% lower protein level within 4 days), but not in similarly-treated scrambled shRNA Tg kidneys (control) [12].

Experimental nephrotoxic nephritis is a model of rapidly progressive glomerulonephritis characterized by: proteinuria; infiltration with macrophages and T-cells; crescent formation in the glomeruli; and Th1 antibody (IgG_2a_) and cytokine (IL12, and INFγ) responses [13]. Macrophages and T-cells play critical roles in the pathogenesis of nephrotoxic nephritis; their numbers peak 7–10 days after anti-GBM antibody administration, and correlate with inflammation and crescent formation [13–19]. Current data suggest that kidney-specific knockdown of STC1 alters the phenotype of nephrotoxic nephritis; where the predominant features include glomerular necrosis/hyalinosis, severe tubular epithelial injury and sloughing, massive cast formation and tubular dilatation. Surprisingly, inflammation is not a dominant feature, as cytokine release and infiltration with macrophages and T-cells are diminished. We propose that absent cytokine release from necrotic tubules may account for the paucity of inflammatory cells within the kidney, consistent with a cross-talk between epithelial cells and the immune system that determines the inflammatory phenotype.

## Experimental Procedures

### Materials

All materials were purchased from Sigma (St Louis, MO) unless stated otherwise. Tie2-Cre plasmid was a gift from Dr. Masashi Yanagisawa, UT Southwestern (Tie2 is endothelium-specific [20]). Sheep anti-mouse GBM antibody was a gift from Dr. Hui Lan (Chinese University of Hong Kong). Goat anti-hSTC1 antibodies and rabbit anti-AQP1 antibodies were purchased from Santa-Cruz Biotechnology, Inc. (Santa Cruz, CA). Rabbit anti-CD3 antibodies, mouse anti-actin and mouse anti-GAPDH were purchased from EMD Millipore (Billerica, MA). Rat anti-F4/80 antibodies were purchased from AbD Serotec (Raleigh, NC). Rabbit anti-sheep IgG and rabbit anti-mouse IgG were purchased from Bethyl Laboratory (Montgomery, TX). Rabbit anti-mouse C3 was purchased from Gene Tex (Irvine, CA). Anti-mouse-IgG,-IgG_1_,-IgG_2a_,-IgG_2b_ and-IgG_3_ antibodies were purchased from Southern Biotechnology Associates (Birmingham, AL). ECL plus reagent was purchased from Fisher Scientific (Pittsburgh, PA).

### Transgenic shRNA Mice

Active shRNA transgenes behave like dominant-negative alleles of the genes of interest [21]. Kidney-specific knockdown of STC1 was achieved as we previously described [12]. STC1 shRNA Tg and scrambled shRNA Tg mice (with similar number of transgene copies) were used. Delivery of Tie2-Cre to the kidney using ultrasound microbubbles, removes a floxed reporter (PGK-EGFP), permitting the expression of the shRNA. And while the shRNA is expressed by endothelial cells, it is transferred to neighboring cells in vivo, in a manner known as non-cell autonomous RNAi [22], permitting kidney-specific knockdown of STC1 in STC1 shRNA Tg kidneys (70% lower protein level within 4 days), but not in similarly-treated scrambled shRNA Tg kidneys (control) [12]. Whole kidney knockdown of STC1 was illustrated using in situ hybridization and immunohistochemistry [12], while non-cell autonomous RNAi transfer was illustrated using transwell co-culture system–where endothelial cells that express STC1 shRNA were placed on the top chamber, and C2C12 cells that normally express high level of native STC1, were placed on the lower chamber; knockdown of STC1 in C2C12 cells was achieved due to transfer of shRNA from endothelial cells [12]. All mice are on C57B/6 genetic background. The investigation conforms to the Guide for the Care and Use of Laboratory Animals published by the US National Institutes of Health, and animal experiments were approved by university ethics review board of Baylor College of Medicine.

### Nephrotoxic Nephritis Model

**Fig 1:**
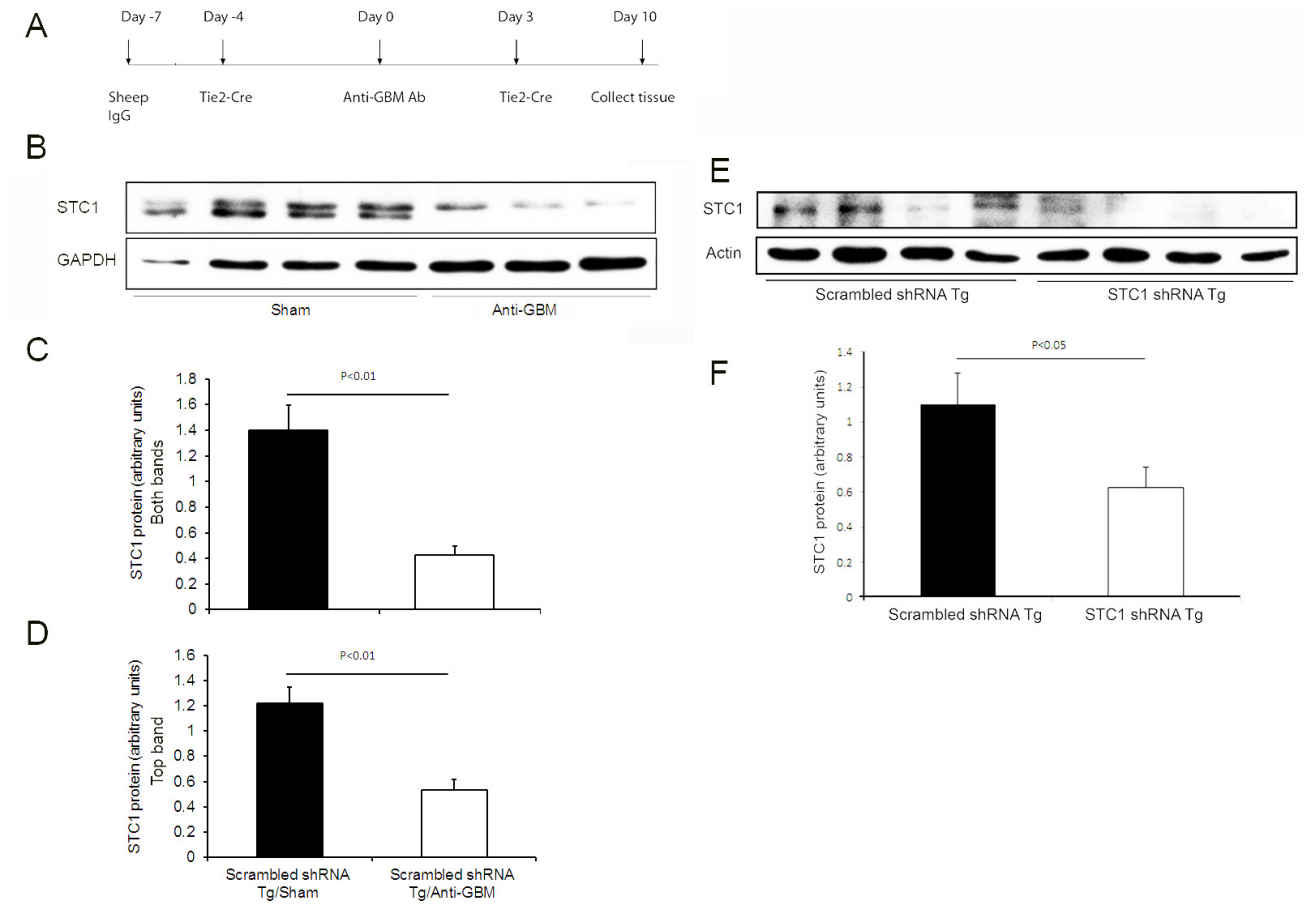
**A**. Timeline of nephrotoxic nephritis experiment. **B**. Representative Western blot depicting STC1 and GAPDH expression in control kidneys 10 days after the administration of anti-GBM or sham. **C & D**. Bar graphs represent the mean ± SEM of STC1 protein/GAPDH in control kidneys 10 days after the administration of anti-GBM (n = 3) or sham (n = 4); **C**, quantitation of both STC1 protein bands relative to GAPDH; **D**, quantitation of top STC1 protein band relative to GAPDH. **E**. Representative Western blot depicting STC1 and actin expression in the kidneys of STC1 shRNA Tg and scrambled shRNA Tg mice, 10 days after the administration of anti-GBM antibody. **F**. Bar graph represents the mean ± SEM of STC1 protein/actin in the kidneys of STC1 shRNA Tg and scrambled shRNA Tg mice, 10 days after the administration of anti-GBM antibody (n = 4).

3–5 months old male mice (25 g) were used; mice were placed on ad-lib food and water intake throughout the experiment. Accelerated nephrotoxic nephritis was induced by subcutaneous “priming” at day -7 with 1 mg/mouse of normal sheep IgG in Freund's complete adjuvant (FCA), followed by delivery of anti-GBM antibody (0.1 mg/g) at day 0 (see Fig 1 for timeline). Tie2-Cre was delivered to the kidneys at day -4 before- and day +3 after- delivery of anti-GBM antibody. Mice were euthanized 10 days after anti-GBM antibody injection, time-point which coincides with peak inflammation [11], and kidneys were harvested for analysis.

### Blood Pressure Measurement and Renal Function Assessment

Systolic blood pressure was recorded in conscious mice at baseline and prior to euthanasia by tail plethysmography using BP2000 blood pressure analysis system (Visitech Systems, Inc., Apex, NC). Urinary protein concentrations were determined by the Bradford method, adapted to a micro-titer plate assay, using bovine serum albumin (Sigma) as standard; Bio-Rad protein assay reagent (Bio-Rad Lab., Hercules, CA) was added to the diluted urine samples, and the absorbance was read at 595-nm wavelength on FLUstar Omega microplate reader (BMG Labtech; Ortenberg, Germany). Urine albumin was measured using albuminuria ELISA kit, as per manufacturer’s instructions (Exocell, Philadelphia, PA). Serum creatinine was measured using BioAssay Systems kit (Hayward, CA), as per manufacturer’s instructions. Please note, compared with capillary electrophoresis [23]- and/or HPLC-based creatinine measurement methods [24], this method overestimates serum creatinine (nearly 2-fold), and thus underestimates glomerular filtration rate by half.

### Immunohistochemistry

Methacarn-fixed kidney sections were dehydrated in graded alcohols and embedded in paraffin blocks using standard techniques; sections were cut, dried and rehydrated before staining. Frozen kidney tissue was embedded in OCT (Optimal Cutting Temperature), and 5 μm sections were stained with: mouse IgG (1:100 dilution); sheep IgG (1:50 dilution); mouse C3 (1:100 dilution). Paraffin-embedded and Methacarn-fixed tissue was stained with: rabbit anti-STC1 (1:300 dilution); rat anti-mouse F4/80 antibody (1:50 dilution); rabbit anti-CD3 antibody (1:200 dilution); or rabbit anti-AQP1 (1:200 dilution). Detection was carried out using fluorescence or peroxidase enzyme-based detection system (Vector Laboratories), as appropriate. Control for labeling was carried out in the presence of non-immune IgG. Photomicrographs were taken using Nikon Eclipse 80i microscope system.

### Morphometric Analysis

Tissue sections were evaluated by a kidney pathologist who was blinded to the experimental protocol. Interstitial volume was determined using a point-counting technique on trichrome-stained sections. Using 1-cm^2^ graded ocular grid viewed at 200X magnification as reference, the interstitial volume was expressed as the percentage of grid points, which lay within the interstitial area of 5 randomly selected fields. Glomeruli with crescent formation, proliferation, hyalinosis/necrosis and sclerosis were expressed as the number of glomeruli manifesting these changes out of the total number of glomeruli surveyed. Glomerular sclerosis, necrosis and/or hyalinosis was determined based on the fraction of the glomerulus stained positively with Periodic Acid Schiff (PAS). Each area was measured by tracking the glomerular tuft aided by computer manipulation using Mac Scope version 6.02 (Mitani Shoji Co., Ltd., Fukui, Japan). The number of glomeruli showing >50% necrosis/hyalinosis or sclerosis was recorded. Total macrophages (F4/80^+^) and T-cells (CD3^+^) infiltrating the glomeruli and interstitium in an entire coronal kidney section were counted, and the results were expressed as total number of cells observed in the glomeruli or interstitium per 10 grids (1-cm^2^ graded ocular grids viewed at 200X magnification).

### Elution of Antibodies from the Kidneys

Kidneys were perfused with saline and stored at -80°C until used. The cortical portions of the kidneys were dissected out by slicing with a razor blade, then mixed with cold PBS (pH 7.4), and homogenized in a 5 mL Dounce homogenizer. This homogenate was spun at 400 x *g* for 7 min at 4°C. The pellet was washed with cold PBS seven times and recovered by centrifugation. Elution of the antibodies from the pellet was carried out as previously described [25]. First, the sediment was suspended in 0.2 M glycine buffer (5 parts buffer:1 part sediment, v/v; pH 2.5), and incubated at room temperature with constant agitation for 2h [25]. The mixture was then spun at 10,000 x *g* for 30 min at 4°C. The supernatant was recovered and immediately brought to pH 7.0 with 0.1N NaOH, followed by dialysis against PBS (several changes). The amount of IgG in the eluate was measured using ELISA. Serum IgG subtypes were measures using ELISA.

### Western Blotting

Lysates, prepared from kidney tissue as previously described [26], were suspended in RIPA buffer containing 1x cocktail of proteinase and phosphatase inhibitors, and centrifuged at 8000 g for 10 min, at 4°C, to remove cell debris. Twenty μg of protein were resolved on 12% SDS-PAGE, transferred to nitrocellulose membrane and incubated with primary antibodies for STC1, GAPDH or actin. After wash with PBS containing 0.1% Tween-20, the membrane was incubated with horseradish peroxidase-conjugated secondary antibody. The bound antibodies were visualized using chemiluminescence. Densitometric analysis of target proteins was performed using NIH ImageJ Software.

### Cytokine PCR Array

Mouse Common Cytokines RT^2^ Profiler^TM^ PCR Array (Qiagen; Frederick, MD), employing a set of optimized PCR primers was used to profile the expression of 84 important cytokine genes using real-time (RT)-PCR on 96-well plates. This array includes tumor necrosis factor (TNF)-α, interferons, interleukins, bone morphogenetic proteins (BMP) and members of the transforming growth factor (TGF)-β family. Also represented are various growth factors (colony-stimulating, fibroblast, insulin-like, platelet-derived, transforming, and vascular endothelial). Total RNA was isolated from whole kidneys, using TRIzol (Invitrogen; Grand Island, NY). 0.5 μg of total RNA from each sample were used for cDNA synthesis (RT2 First Standard kit; Qiagen), followed by RT-PCR, as per manufacturer’s instruction. Data represent the mean ± SEM of three independent determinations, and results were normalized to 3 house-keeping genes included in the array [B2m; GAPDH; HSP90ab1). Two-fold change was set as the threshold for decreased or increased expression of a particular gene.

To examine cytokines and growth factors expression in vitro, immortalized mouse proximal tubules cells (TKPTS [27]) were treated with H_2_O_2_ (0.6 mM, for 2h); cells were scraped and collected by centrifugation (1000 g for 5 min), and RNA was isolated using TRIzol. One microgram of total RNA from each sample was used for cDNA synthesis, using iScript cDNA synthesis kit (Bio-Rad Lab. Inc.), followed by RT-PCR, and the abundance of select cytokines and growth factors (CCL17, CCL19, CX3CL1, Gpi1, Mif, Spp1, VEGF) was determined. Data were normalized to the house-keeping gene actin amplified in the same reaction. Primers used: CCL17: sense 5’-TGCTGCCTGGATTACTTC-3’, antisense 5’-ACACTCCACTGAGGTCTTA-3’; CCL19: sense 5’-CAAGAACAAAGGCAACAG-3’, antisense 5’-CTTCTGGTCCTTGGTTTC-3’; CX3CL1: sense 5’-TGACGAAATGCGAAATCA-3’, antisense 5’-TTAGCTGATAGCGGATGA-3’; Gpi1: sense 5’-TGTCTCTAACATTGATGG-3’, antisense 5’-CGATTATAAAGAGGGAAGT-3’; Mif: sense 5’- TACATCAACTATTACGACAT-3’, antisense 5’-TAAACACAGAACACTACG-3’; Spp1: sense 5’- AATGCTGTGTCCTCTGAA-3’, antisense 5’-TCGTCATCATCATCGTCAT-3’; VEGFA: sense 5’- GACTATTCAGCGGACTCA-3’, antisense 5’-AAGAACCAACCTCCTCAA-3’; Gusb: sense 5’- TCTGTGACTGACTACTAC-3’, antisense 5’-GAATCCTCGTGCTTATTG-3’; actin: sense 5’-ATCTTCCGCCTTAATACT-3’, antisense 5’-GCCTTCATACATCAAGTT-3’.

### Statistical Analysis

Data are expressed as means ± SEM and are compared by one way ANalysis Of VAriance (ANOVA) for three or more groups, or unpaired t-test. Statistical significance of difference was defined by a *p*-value of less than 0.05.

## Results

### Kidney-Specific Knockdown of STC1 Alters the Phenotype of Nephrotoxic Nephritis

While STC1 Tg mice are protected from nephrotoxic nephritis [11], it was unclear whether the protection is mediated through local effects of STC1 in the kidney, or extra-renal effects due to high circulating levels of rSTC1. To determine the impact of kidney-specific knockdown of STC1 on the phenotype nephrotoxic nephritis, we utilized STC1 shRNA Tg and scrambled shRNA Tg mice, which we recently described in details [12]. Delivery of Tie2-Cre to the kidney using ultrasound microbubbles, removes a floxed reporter (PGK-EGFP), permitting the expression of shRNA and kidney-specific knockdown of STC1 in STC1 shRNA Tg kidneys (70% lower protein level within 4 days), but not in similarly-treated scrambled shRNA Tg kidneys (control) [12]. Since activation of STC1 shRNA expression leads to knockdown of STC1 within 4 days, delivery of Tie2-Cre was timed for day -4 before administration of anti-GBM antibody, and day 3 after administration of anti-GBM antibody. The experiment was terminated at day 10, as the mice appeared moribund.

Surprisingly, STC1 expression decreases after nephrotoxic nephritis in control kidneys relative to sham-treated controls (Fig 1). However, at the point of termination of the experiment (day 10), and following nephrotoxic nephritis, STC1 protein level in STC1 knockdown kidneys was 54% of controls (Fig 1).

**Fig 2:**
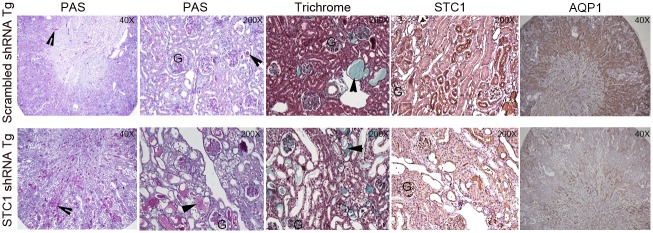
Kidney morphology, fibrosis and the expression of STC1 and AQP1 after nephrotoxic nephritis in STC1 knockdown kidneys (STC1 shRNA Tg), compared with control kidneys (scrambled shRNA Tg). Kidney tissue was harvested 10 days after the administration of anti-GBM antibody and stained with Periodic Acid Schiff’s (PAS), Masson’s trichrome, STC1 and AQP1; 40X-200X magnification. Arrowheads point to casts; G, denotes glomerulus.

Consistent with previous observations we made in WT mice [11], administration of anti-GBM Ab to control mice resulted in crescentic glomerulonephritis, associated with intra- and extra-capillary mononuclear cell infiltration, mild-to-moderate tubular dilatation, mild-to-moderate hyaline cast formation and glomerulosclerosis (Fig 2 and Table 1). Interestingly, administration of anti-GBM Ab following kidney-specific knockdown of STC1 resulted in: severe proximal tubular epithelial injury and sloughing, as determined by loss of AQP1 staining (Fig 2); massive cast formation and severe tubular dilation; 7-fold greater number of glomeruli with necrosis/hyalinosis; and greater expansion of the tubulointerstitial compartment (Fig 2 and Table 1).

**Table 1:** Morphometric analysis of nephrotoxic nephritis in STC1 knockdown kidneys vs. controls. Table shows total number of glomeruli surveyed. Of these glomeruli, the number of glomeruli displaying: cell proliferation; hyalinosis/necrosis; crescent formation; and sclerosis were counted. Also listed, tubulointerstitial (TIN) expansion grade (see methods) and the total number of casts counted in 3 representative kidneys in each group (at 400X magnification).

	Total glomeruli	Glomerular cell proliferation	Glomerular hyalinosis/necrosis	Crescentic glomeruli	Sclerotic glomeruli	TIN grading	Tubular cast, cortex	Tubular cast, medulla
**Scrambled shRNA Tg, anti-GBM**	**107±7.6**	**1.2±0.4**	**6.3±4.6**	**0.5±0.3**	**0.5±0.3**	**0.5±0.3**	**20.3±6.0**	**50.8±16.0**
**STC1 shRNA Tg, anti-GBM**	**76.8±3.7**	**1.3±0.2**	**37.8±10.8**	**2.5±1.0**	**1.0±1.0**	**2.8±0.8**	**177±28.6**	**Too many to count**
**P value**	**P<0.01**	**NS**	**P<0.05**	**P = 0.5**	**NS**	**P<0.05**	**P<0.001**	

**Fig 3:**
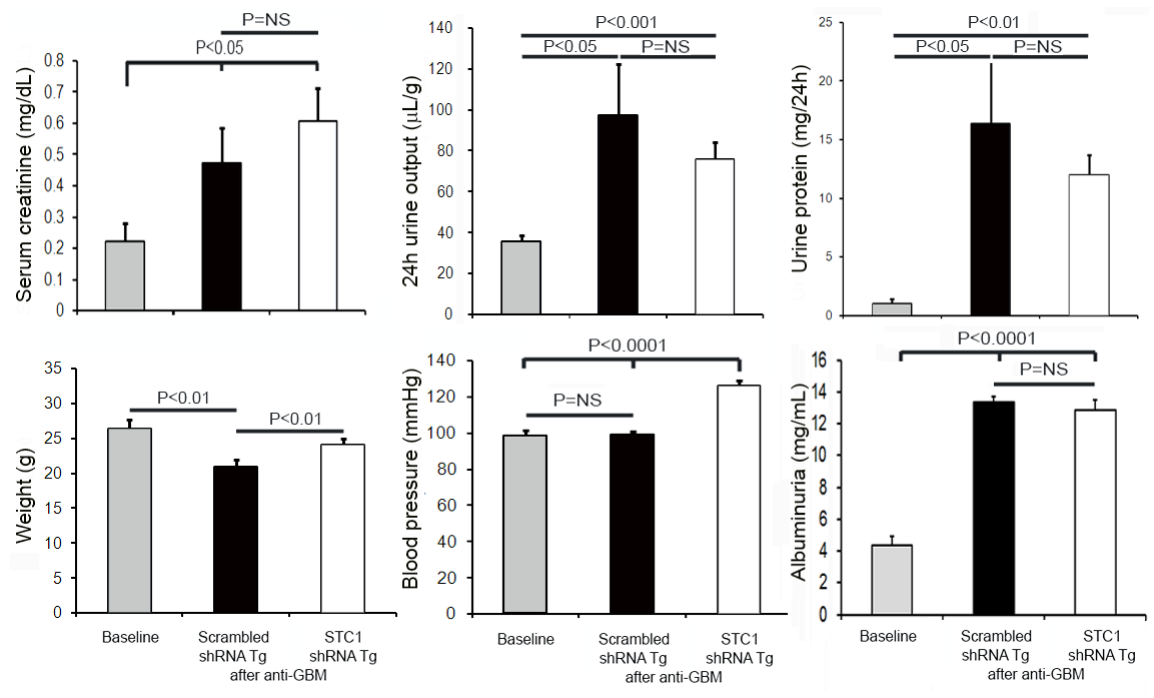
Serum creatinine, urine output, urine protein, albuminuria, weight and blood pressure after nephrotoxic nephritis in STC1 knockdown (STC1 shRNA Tg) compared with control mice (scrambled shRNA Tg). Following nephrotoxic nephritis and compared to baseline, serum creatinine was numerically higher in STC1 knockdown kidneys relative to controls (3-fold vs. 2.5-fold); urine output and proteinuria where numerically lower (2.1-fold vs. 2.85-fold and 12-fold vs. 17-fold, respectively), but the differences between the groups were not statistically significant; albuminuria was increased to a similar degree. Blood pressure and weight were higher following nephrotoxic nephritis in STC1 knockdown kidneys compared to control kidneys.

**Fig 4:**
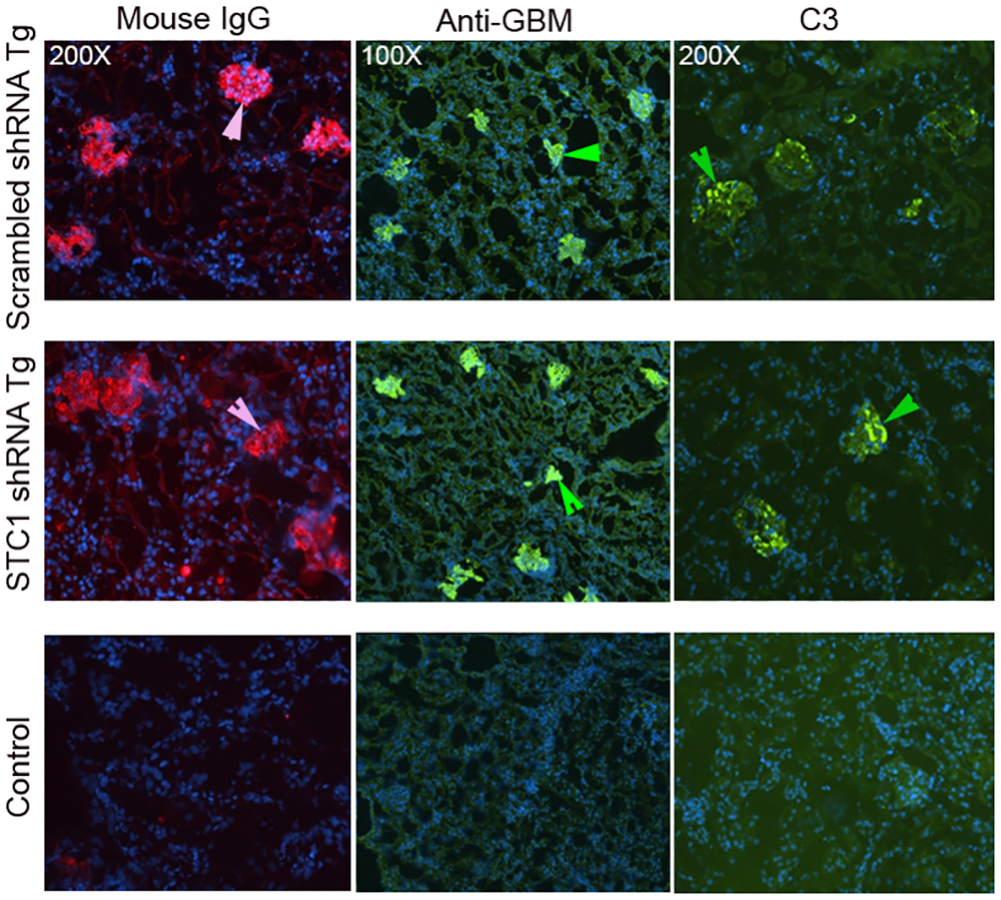
Equal binding of sheep IgG to GBM, and deposition of mouse IgG and C_3_ in STC1 knockdown kidneys and control kidneys. Ten days after anti-GBM Ab injection, kidney sections were labeled with: rabbit anti-mouse IgG antibodies; rabbit anti-sheep IgG antibodies; or rabbit anti-mouse C_3_ antibodies. Equal staining for sheep anti-mouse GBM antibody and deposition of mouse IgG and C_3_ in both STC1 knockdown and control kidneys is illustrated; representative images are shown; 100X-200X magnification. Arrowheads point to glomeruli; lower panels show negative staining in wild type kidneys (control).

**Fig 5:**
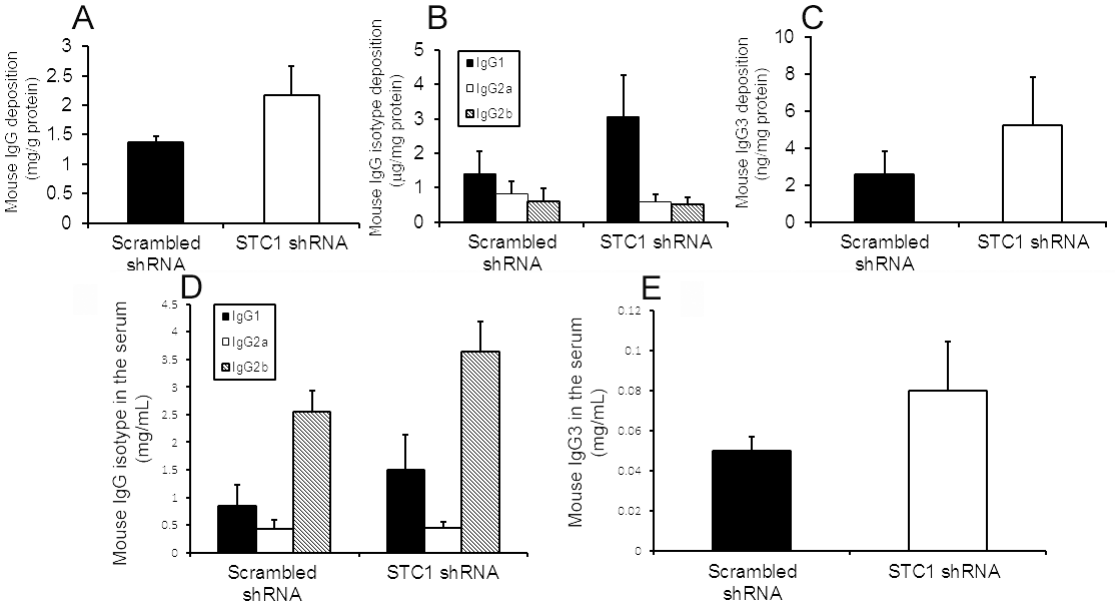
Equal deposition of mouse IgG and IgG isotypes after nephrotoxic nephritis in STC1 knockdown and control kidneys. Bar graphs depict eluted mouse IgG (**A**) and IgG isotypes (**B & C**) from STC1 knockdown and control kidneys after nephrotoxic nephritis. Data represent the means ± SEM of 7 independent determinations for STC1 shRNA Tg and 5 independent determinations for scrambled shRNA Tg. **Equal mouse antibody response to sheep IgG after nephrotoxic nephritis in STC1 knockdown and control mice**. Bar graphs depict mouse serum IgG isotypes (**D & E**) after nephrotoxic nephritis in STC1 knockdown and control mice. Data represent the means ± SEM of 7 independent determinations for STC1 shRNA Tg and 5 independent determinations for scrambled shRNA Tg.

While serum creatinine was numerically higher after nephrotoxic nephritis in STC1 knockdown kidneys relative to controls (3-fold vs. 2.5-fold), and urine output and proteinuria where numerically lower (2.1-fold vs. 2.85-fold and 12-fold vs. 17-fold, respectively), the differences were not statistically different between the groups. However, consistent with worse kidney injury, blood pressure and weight were higher following nephrotoxic nephritis in STC1 knockdown kidneys compared with control kidneys (Fig 3). We attribute the lack of differences (statistically significant) in functional parameters between the two groups to early termination of the experiment, as some of the mice appeared moribund. Control studies indicated equal deposition of sheep anti mouse-GBM antibody, mouse C_3_, mouse IgG and IgG subtypes in STC1 knockdown and control kidneys, and equal mouse antibody response to sheep IgG (Figs 4 and 5); consistent with equivalent anti-GBM-mediated kidney injury and similar host antibody and complement responses.

### Decreased Infiltration with T-Cells and Macrophages following Nephrotoxic Nephritis in STC1 Knockdown Kidneys Compared with Control

**Fig 6:**
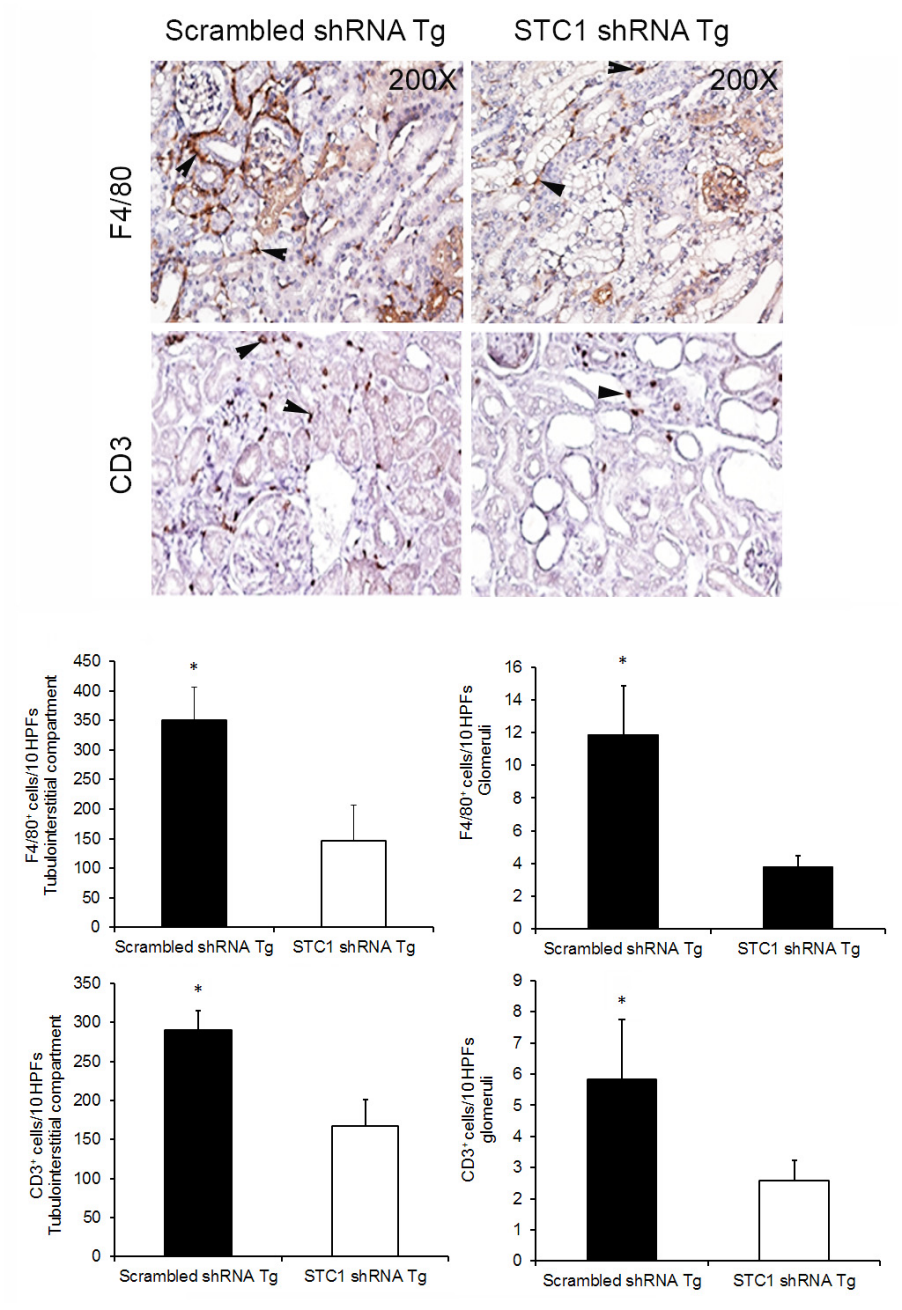
Diminished infiltration with macrophages and T-cells after nephrotoxic nephritis in STC1 knockdown kidneys, compared with controls. Top panels show representative images for F4/80^+^ (macrophages and dendritic cells) and CD3^+^ (lymphocytes) staining; arrowheads point to positively stained cells; 200X magnification. Bar graphs depict macrophage and T-cell count in the glomeruli and interstitial compartment (see methods); data represent the means and ± SEM of 5–7 independent determinations. *, P<0.05.

We have previously shown rSTC1 stabilizes endothelial barrier function, and diminishes trans-endothelial migration of leukocytes; while transgenic overexpression of STC1 in mice diminishes kidney inflammation in the context of nephrotoxic nephritis [11]. Therefore, we hypothesized that kidney inflammation would increase in STC1 knockdown kidneys following nephrotoxic nephritis. Surprisingly, 10 days after anti-GBM Ab injection, time point reported to correlate with peak infiltration with macrophages and T-cells in experimental mouse nephrotoxic nephritis [28,29], there were fewer T-cells (CD3^+^) and macrophages (F4/80^+^) in STC1 knockdown kidneys compared with control kidneys (Fig 6).

### Decreased Expression of Cytokines and Growth Factors after Nephrotoxic Nephritis in STC1 Knockdown Kidneys

Nephrotoxic nephritis is associated with increased expression of cytokines/lymphokines, including interleukin-1β (IL-1 β), TNF-α, TGF- β and macrophage chemotaxis protein (MCP)-1 [30–36]. Tubular epithelial cells can release cytokines/chemokines/growth factors and recruit inflammatory cells and mesenchymal stem cells to the site of injury [37–40]; these in turn may participate in the inflammatory response and/or promote tubular regeneration and recovery. Because of the paucity of inflammatory cells we observed after nephrotoxic nephritis in STC1 knockdown kidneys, we hypothesized that STC1 knockdown kidneys produced less cytokines/chemokines and growth factors due to the severe tubular injury and cell necrosis they sustained; hence the low number of T-cells and macrophages. To validate this hypothesis, we employed cytokine PCR array to examine the expression of 84 cytokines and growth factors in STC1 knockdown and control kidneys after nephrotoxic nephritis. The data revealed reduced expression of 78 (out of 84) genes in STC1 knockdown kidneys relative to control kidneys (Table 2), with notable statistically significant reduction in the expression of CCL12 (21-fold), CX3CL1 (3-fold), CCL17 (6-fold) and CCL19 (20-fold). The underlined genes will be further examined in cultured proximal tubule cells. Six genes where expressed at higher level in STC1 knockdown kidneys relative to control kidneys; however, the differences were not statistically significant. These include: macrophage migration inhibitory factor (MIF); glucose phosphate isomerase 1 (Gpi1); osteopontin (Spp1); and vascular endothelial growth factor (VEGF). The underlined genes will also be examined in cultured proximal tubule cells.

**Table 2:** Cytokine array. Mouse Common Cytokines RT^2^ Profiler^TM^ PCR Array (using total RNA representing whole kidney) was used to survey the expression of 84 important cytokine genes and growth factors after nephrotoxic nephritis in STC1 knockdown kidneys and controls. Middle column shows fold decrease in gene expression in STC1 knockdown kidneys relative to controls. P values highlighted in bold correspond to genes expressed at significantly lower levels after nephrotoxic nephritis in STC1 knockdown kidneys relative to controls.

Gene Symbol	Fold Regulation	p-value
Adipoq	-3.1172	0.326691
Bmp2	-2.7426	0.325054
Bmp4	-1.31	0.153817
Bmp6	1.95	0.208209
Bmp7	-1.11	0.661736
Ccl1	-3.0366	0.32269
Ccl11	-3.9557	0.351233
Ccl12	-21.1337	**0.02145**
Ccl17	-6.3584	**0.049754**
Ccl19	-20.5035	**0.006242**
Ccl2	-8.0673	0.164969
Ccl20	-3.7133	0.230301
Ccl22	-3.758	0.336398
Ccl24	-3.3707	0.336839
Ccl3	-4.9296	0.34317
Ccl4	-5.3752	0.297101
Ccl5	-6.1528	0.063276
Ccl7	-10.0101	0.077521
Cd40lg	-3.1558	0.32846
Cd70	-3.8218	0.348712
Cntf	-2.2442	0.372276
Csf1	-1.46	0.613587
Csf2	-1.41	0.480319
Csf3	-3.831	0.348898
Ctf1	-2.7083	0.330676
Cx3cl1	-2.8874	**0.019457**
Cxcl1	1.14	0.543511
Cxcl10	-4.8673	0.138326
Cxcl11	-4.0331	0.352536
Cxcl12	-1.98	0.105972
Cxcl13	-2.2908	0.357902
Cxcl3	-4.4049	0.343487
Cxcl5	-5.2756	0.144523
Cxcl9	-4.3341	0.329792
Fasl	-3.526	0.341642
Gpi1	1.31	0.071687
Hc	-6.4098	0.292892
Ifna2	-3.9177	0.350554
Ifng	-3.9839	0.35172
Il10	-4.2003	0.355028
Il11	-3.7172	0.346475
Il12a	-4.1803	0.354752
Il12b	-4.2461	0.355642
Il13	-4.0032	0.352046
Il15	-3.389	0.095815
Il16	-3.4004	0.239249
Il17a	-2.5413	0.284654
Il17f	-3.5598	0.343352
Il18	-3.7085	0.253989
Il1a	-3.2813	0.360731
Il1b	-5.36	0.275903
Il1rn	-2.4578	0.461784
Il2	-4.0365	0.352591
Il21	-3.7482	0.347165
Il22	-4.6533	0.360082
Il23a	-4.3702	0.357176
Il24	-4.1566	0.354416
Il27	-3.6187	0.344117
Il3	-3.607	0.34382
Il4	-4.0902	0.353434
Il5	-3.6651	0.34526
Il6	-3.4233	0.338567
Il7	-3.5459	0.35716
Il9	-3.9052	0.350324
Lta	-3.7784	0.347815
Ltb	-8.0077	0.218989
Mif	1.24	0.441875
Mstn	-4.0292	0.352474
Nodal	-3.1878	0.329858
Osm	-3.6644	0.345244
Pf4	-3.5116	0.327895
Ppbp	-2.7638	0.378108
Spp1	1.03	0.959899
Tgfb2	-3.1965	0.287718
Thpo	-1.7	0.246895
Tnf	-3.57	0.342763
Tnfrsf11b	-4.33	0.356678
Tnfsf10	-4.41	0.076277
Tnfsf11	-3.99	0.351837
Tnfsf13b	-4.78	0.131674
Vegfa	1.14	0.643747
Xcl1	-6.4375	0.265082

### Cultured Proximal Tubule Cells Express Cytokines and Growth Factors in Response to H_2_O_2_ Stress

After nephrotoxic nephritis, our data revealed fewer inflammatory cells and diminished expression of cytokines in STC1 knockdown kidneys relative to control kidneys. Because the phenotype of nephrotoxic nephritis in STC1 knockdown kidneys was characterized by tubular epithelial necrosis and not inflammation, we hypothesized that epithelial cells constitute an important source for cytokines/chemokines and growth factors.

**Fig 7:**
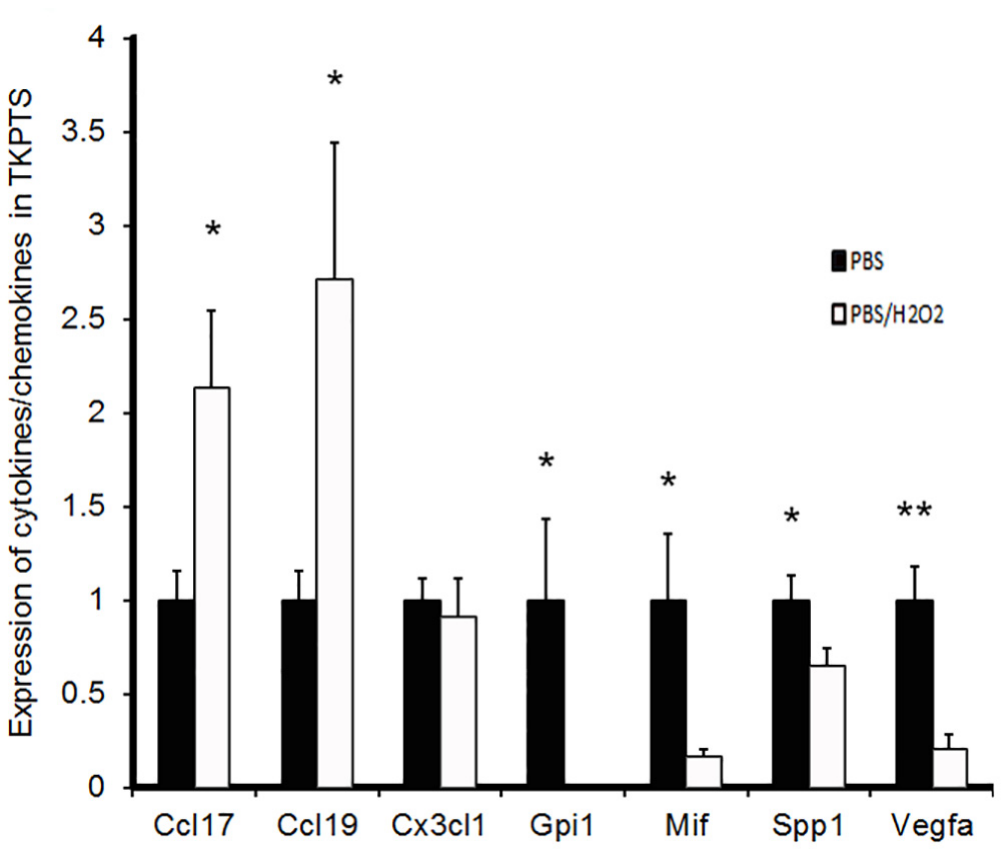
Differential expression of cytokines/chemokines/growth factors by proximal tubule cells in response to H_2_O_2_ stress. Immortalized mouse proximal tubules cells (TKPTS) were treated with vehicle or H_2_O_2_; mRNA for select cytokines/chemokines (CCL17, CCL19, CX3CL1 and Mif) and growth factors (Gpi1, Spp1 and VEGF) were quantitated using RT-PCR (see methods). Data are normalized to actin, and represent the mean ± SEM of 6 independent determinations. *, P<0.05; **, P<0.01.

To that end, we subjected cultured proximal tubule epithelial cells to mild H_2_O_2_ stress as an inflammatory mimic [41], and determined the expression of a number of cytokines/chemokines that were altered after nephrotoxic nephritis in STC1 knockdown kidneys relative to control kidneys. These included 3 of 4 chemokines that were significantly attenuated after anti-GBM in STC1 knockdown kidneys relative to control kidneys (CCL17, CX3CL1 and CCL19), and 4 of 6 genes that were increased after nephrotoxic nephritis in STC1 knockdown kidneys relative to control kidneys (MIF, Gpi1, Spp1, and VEGF). As shown in Fig 7, all genes tested were expressed in proximal tubule cells. Mild H_2_O_2_ stress induced the expression of pro-inflammatory chemokines CCL17 and CCL19; the expression of chemokine CX3CL1 was unchanged, while the expression of the pro-inflammatory protein MIF was reduced. Of interest, the expression of pro-survival proteins Gpi1, Spp1 and VEGF was reduced. Thus, in agreement with previously published reports [37–40], our *in vivo and in vitro* data suggest that tubular epithelial cells respond to stress and injury by releasing cytokines and growth factors.

## Discussion

STC1 is a paracrine/intracrine protein (an intracellular-acting, extracellular signaling protein [6]). It is released to the extracellular milieu [2], binds to a cell-surface protein [3], followed by internalization and targeting to the inner mitochondrial membrane [4,5]. Based on cumulative data, STC1 appears to function as a naturally occurring anti-inflammatory and cytoprotective protein [9,10]. In the context of inflammation, it acts through a number of novel mechanisms that affect the endothelium and macrophages. In macrophages STC1: decreases intracellular calcium signal [9], diminishing macrophage mobility and its response to chemoattractants [9]; and inhibits macrophage function through suppression of superoxide generation [8]. In the endothelium, STC1: suppresses superoxide generation [10]; inhibits pro-inflammatory signaling through NF-κB and JNK [7]; blocks cytokine-induced changes in tight junction proteins [7]; preserves endothelial barrier functions [7]; and dose-dependently decreases trans-endothelial migration of macrophages and T-cells *in vitro* [10] and *in vivo* [23]. Thus, STC1 provides potent anti-inflammatory actions.

In addition, recent data from our lab suggest that STC1 is essential for the survival of proximal tubule cells under normal physiologic conditions, as conditional and kidney-specific knockdown of STC1 increases the production of reactive oxygen species, and leads to proximal tubular injury and moderate kidney failure (50% reduction in GFR) [12]. STC1 expression is widely distributed in the kidney, and the unique susceptibility of the proximal tubules to STC1 knockdown may be related to their high metabolic rate and immediate exposure to the glomerular filtrate which may contain substances that promote oxidant injury [12].

Kidney injury in nephrotoxic nephritis is driven by inflammation and is characterized by infiltration with T-cells and macrophages, Th1 antibody (IgG_2a_) and cytokine (IL12, and INFγ) responses [13]. Macrophages and T-cells play important roles in the pathogenesis of nephrotoxic nephritis, and their numbers correlate with inflammation and crescent formation [13–19]. Notwithstanding the proximal tubular injury and moderate kidney failure that we expected after knockdown of STC1 in the kidney, absence of STC1 was expected to promote inflammation after nephrotoxic nephritis. Surprisingly, inflammation was not a prominent feature of nephrotoxic nephritis in STC1 knockdown kidneys, as the expression of cytokines/chemokines and infiltration with macrophages and T-cells were diminished. Instead, nephrotoxic nephritis following kidney-specific knockdown of STC1 was associated with glomerular necrosis/hyalinosis, severe tubular epithelial injury and sloughing, massive cast formation, and kidney failure. We speculate that the prominent tubular injury phenotype we observe may be related to higher susceptibility of epithelial cells to the nephrotoxic injury in the absence of STC1-mediated anti-oxidant defenses. Similarly, epithelial cells in the glomeruli may share this susceptibility; and hence the glomerular necrosis/hyalinosis after nephrotoxic nephritis in STC1 shRNA Tg kidneys. Of interest and previously unrecognized, nephrotoxic nephritis is associated with diminished expression of STC1 in control kidneys. In light of recent insight about the importance of STC1 for the survival of proximal tubule cells under normal physiologic conditions [12], this finding may be critical to understanding the pathophysiology of tubular injury in the context of nephrotoxic nephritis. The mechanism of STC1 suppression in the context of nephrotoxic nephritis remains unclear.

We hypothesized that tubular epithelial cells produce cytokines/chemokines and participate in the recruitment of inflammatory cells to the site of injury; however, due to the severe tubular necrosis we observe after nephrotoxic nephritis in STC1 knockdown kidneys, there is diminished cytokine/chemokine release from epithelial cells, and as a result, decreased recruitment of inflammatory cells to the kidney in response to nephrotoxic nephritis. Moreover, recovery of these kidneys would be hampered if the epithelial cells were to release growth and survival factors. Indeed, consistent with our hypothesis, survey of 84 genes that code for cytokines/chemokines and growth factors, revealed reduced expression of 78/84 cytokines/chemokines and growth factors, after nephrotoxic nephritis in STC1 knockdown kidneys, relative to control kidneys.

Because cytokines/chemokines and growth factors may have different cellular origins within the kidney, and to validate the hypothesis that tubular epithelial cells produce cytokines/chemokines and participate in the inflammatory response, we subjected cultured proximal tubular epithelial cells to H_2_O_2_ stress, an inflammatory mimic [41], and determined the expression of a number of cytokines/chemokines and growth factors that were significantly altered after nephrotoxic nephritis in STC1 knockdown kidneys relative to control kidneys. Our data revealed expression of all cytokines/chemokines and growth factors examined in cultured tubular epithelial cells, and differential response of these cytokines/chemokines/growth factors to H_2_O_2_ stress.

Thus, our *in vivo* and *in vitro* data are in agreement with previously published reports that suggest release of cytokines/chemokines and growth factors by tubular epithelial cells in response to stress/injury [37–40]. These cytokines/chemokines/growth factors in turn participate in the inflammatory response and aid in the recovery from stress and injury; absent cytokines/chemokines release from tubular epithelial cells after nephrotoxic nephritis in STC1 knockdown kidneys may explain the paucity of inflammatory cells, while diminished expression of growth factors may hamper the recovery from injury.

Indeed, all 4 chemokines which display significantly reduced expression after nephrotoxic nephritis in STC1 knockdown kidneys relative to control kidneys [CCL12 (21-fold); CXCL1 (3-fold); CCL17 (6-fold); CCL19 (20-fold)] are important for the recruitment of macrophages and lymphocytes. Mouse CCL12 (also known as monocyte chemotactic protein 5; MCP-5) is similar in function to human MCP-1; it is highly induced in macrophages, attracts eosinophils, monocytes and lymphocytes and plays a critical role in the development of inflammatory allergic responses [42]. Human CX3CL1 (also known as fractalkine; or mouse neurotactin) is a 373-amino acid protein with an extended mucin-like stalk and a chemokine domain. The stalk permits binding to the surface of some cells (e.g., endothelial), while the chemokine domain may remain tethered to the stalk, or exist as a soluble 90 kD variant [43]. The soluble form exerts chemotaxis for T-cells and monocytes, while the cell-bound chemokine is normally expressed on the surface of activated endothelial cells and promotes the adhesion of leukocytes [44]. CCL17 (also known as thymus and activation regulated chemokine; TARC) induces T-cell chemotaxis through interaction with CCR4 receptor [45]. While CCL19 regulates lymphocyte homing through interaction with CCR7 receptor [46].

In conclusion, nephrotoxic nephritis after kidney-specific and conditioned knockdown of STC1 assumes a phenotype characterized by tubular and glomerular necrosis/hyalinosis. Contrary to our initial hypothesis, where aggravated inflammation was expected (more T-cells, macrophages and cytokines/chemokines) after nephrotoxic nephritis in STC1 knockdown kidneys, we observed fewer inflammatory cells and cytokines/chemokines, and we attribute these findings to the diminished expression of cytokines/chemokines and growth factors by the severely injured tubular epithelial cells, consistent with active participation of tubular epithelial cells in the immune response, in a disease that is considered fundamentally glomerular; a similar response is to be expected in other inflammatory diseases of the kidney.

## Abbreviations

STC1: stanniocalcin-1
Tg: transgenic
GBM: glomerular basement membrane
Tie2: tyrosine-protein kinase receptor
TNF: tumor necrosis factor
BMP: bone morphogenetic proteins
TGF: transforming growth factor
IL: interleukin
MCP: macrophage chemotaxis protein
PAS: Periodic Acid Schiff; C_3_, complement component 3
FCA: Freund's complete adjuvant
CD3: cluster of differentiation-3, a marker of T-cells
CCL12: Chemokine (C-C motif) ligand 12
MIF: macrophage migration inhibitory factor
CX3CL1: a chemokine (fractalkine)
CX3CR1: receptor specific for CX3CL1
TIN: tubulointerstitium.
